# Book Reviews

**Published:** 1802-12-01

**Authors:** 


### CRITICAL ANALYSIS

### OF THE

### RECENT PUBLICATIONS

### ON THE DIFFERENT BRANCHES OF

### PHYSIC, SURGERY, & MEDICAL PHILOSOPHY.

---

*A Description of the Egyptian Ophthalmia, and the Method of Treatment. By* Cit. SAVARESI, *Physician in Ordinary to the (French) Army of the East. (Translated from the Memoires sur l'Egypte, Tom. 2.)*

THE Egyptian Ophthalmia attacks perſons in perfect health; it is therefore difficult to be guarded againſt, but in general the diſeaſe is entirely local; when however it aſſumes an unfavourable character, the pulſe is affected, and it may then be conſidered as an internal inflammation. The progreſs of this diſorder is rapid, and the termination often protracted to a great length of time; if a period is not put to it in ſeven or eight days, it frequently laſts one or two months. From attentive obſervation, I have found that the left eye is more frequently affected than the right, and the ſevereſt ſymptoms appear to be periodical. Sometimes when diarrhœas, dyſenteries, or tertian fevers ſupervene, they carry off all traces of the ophthalmia. When a cure is not effected, the diſeaſe, after reſiſting the moſt active and potent remedies, terminates in amauroſis, in dimneſs of ſight, or even in an entire loſs of the globe of the eye.

*Causes.*—I believe the Egyptian Ophthalmia to be endemic, and the following are my reaſons for holding this opinion.

The champaign country of Egypt conſiſts of immenſe plains, over which the day-light is very bright and intenſe, the ſoil is dry, friable, and burning, particularly in ſummer, it is argillaceous and chalky, chalky, and contains perfect nitre, foda, and fea falt; the days are ferene and fultry, and the nights cool, damp, and cloudy. It is evident that an union of thefe circumftances forms a fufficient phyfical caufe of injury to the eyes of animals expofed to them, by ftimulating in an exceffive degree; hence arifes a defluxion of humours to the part, whereby the equilibrium of its natural tone is broken, and thus either by an increafe or diminution of tone, the *sthenic* or the *asthenic* ophthalmia is produced.

Nothing indeed more ftrikes the traveller into Egypt, than the prodigious number of blind, or perfons affected with difeafes of the eyes, which he every where meets with; for both rich and poor, the inhabitants of towns and of the country, are all equally expofed to this calamity. Hiftory likewife informs us, that even feveral of the Pharoahs were blind at their death. The other animals are not lefs fubject to difeafes of the eyes than the inhabitants themfelves; the greater number of the dogs are either entirely blind or at leaft dim-fighted, and many of the affes, oxen, horfes, and camels have fpots or other flight affections on the eye.

From all thefe facts I am led to conclude, that the ophthalmia is endemic in the countries watered by the Nile, and is particularly virulent in the hot feafon, that is, from the beginning of fummer to the end of autumn.

Some perfons have pretended that thofe people, who, like the Egyptians, feed principally or very largely on rice, were fubject to this malady. Were this the cafe, the Italians, and efpecially the inhabitants of Lombardy, fhould be infefted with the complaint; but as this does not happen with them, nor with many other nations who employ rice as their chief food, no other refutation of this opinion is required.

Among the caufes of ophthalmia in Egypt, the nitrous duft or earth which fo much abounds there is ufually adduced. By this term muft be underftood thofe neutral falts arifing from the combination of the nitric acid with a fixed alkali or a fimple earth. But as all thefe, except nitrat of potafh, abforb the humidity of the atmofphere, they cannot remain in their neutral ftate under the form of duft. Befides, as the nitrous acid has a ftronger affinity with potafh than with foda or the primitive earths (except barytes) the term nitrous duft can here only apply to nitrat of potafh; and this falt, as I fhall fhew, does not injure the organs of fight.

Clay and chalk are earths which are very extenfively fpread over the whole of Egypt, and experience fhews that thefe will very certainly produce ophthalmia. To prove it, I introduced them in fine powder within the eyelids of feveral dogs, who all became almoft blind the day after. On the other hand I found that nitrat of potafh employed in the fame manner produced no inconvenience.

Almoft all the mafons of Egypt have complaints in their eyes, becaufe, as their mode of working is unfkilful and inconvenient, they are conftantly handling lime, and breathing an atmofphere full of particles of chalk, clay, or calcareous earth.

*Nosology*.—This ophthalmia is either fthenic or afthenic, that is to fay, arifing from excefs or deficiency of tone. Of the fthenic, there is is only one ſpecies, namely, the inflammation of the bulb of the eye; the aſthenic includes two ſpecies, the inflammation of the tarſus, and of the conjunctiva. Each of theſe three ſpecies is marked by very characteriſtic ſymptoms.

In the inflammation of the bulb of the eye the following are the moſt prominent ſymptoms; the eyelids are red and inflamed, and open with much difficulty, and at the ſame time the globe of the eye is inſupportably painful within; the ſmall veſſels of the conjunctiva are ſo overloaded with blood as to form a membranous pellicle which ſurrounds the eye.

The ſight is dim, cloudy, and ſometimes altogether loſt, light is inſupportable, a purulent diſcharge ſupplies the place of tears, and the patients often complain of the feeling of ſmall ſtones pricking the eyes, and a piece of cloth covering them.

The inflammation of the tarſi is attended with ſwelling of the upper eyelids, which grow pale and relaxed, and open with difficulty; light produces a diſagreeable ſenſation, the tarſi are painful, inflamed, and watery.

In the inflammation of the conjunctiva the light is inſupportable, the pain acute, the ſight obſcured, and the eye watery.

*Treatment.*—In all the varieties of this ophthalmia I began by purging the patients indiſcriminately with an ounce of ſulphat of magneſia, after which I directed my attention to the peculiar indications of the caſe.

The ſthenic ophthalmia requires the attention of a careful and experienced phyſician, becauſe the cure depends on the activity of the remedies firſt employed. In this caſe much advantage is derived from a bliſter to the nape of the neck, and local bleeding from the temporal artery or jugular vein; theſe ſhould never be omitted, and in an hour after the bleeding, the diſeaſe generally abates in a remarkable degree, the ſpaſm and acute pain diminiſh, and are ſcarcely perceptible on the ſucceeding day. Sometimes however the relief is not quite ſo ſpeedy, and the diſorder remains attended with a ſlight febrile agitation, which requires for its removal general bleeding and purgatives. The regimen ſhould be moderate; for drink, the patient is preſcribed barley-water acidulated with cream of tartar; and an anodyne reſolvent collyrium is uſed, compoſed of laudanum and a decoction of ſaffron. This mode of treatment ſhould be purſued till the ſwelling diminiſhes and the eyelids become inverted, with a certain increaſe of bulk, an appearance which conſtantly takes place, and is owing to the debility and relaxation of the veſſels. When this happens, a ſaponaceous collyrium, compoſed of ſoap diſſolved in ſpirit of wine, is preſcribed, and under its uſe the eyelids regain their natural poſition and open freely, exhibiting the cornea beneath them, which is now either ſlightly red, or elſe ſpotted; in the former caſe, the topical application of cold water and vinegar is highly uſeful; in the latter, a dry collyrium, compoſed of ſugar candy, alum, and nitre is applied, which removes the ſpots in a few days. Under the uſe of theſe topical and general remedies, a cure is generally performed in one or two month's month's time; but if this does not take place within this period, there is reaſon to deſpair of ever reſtoring the parts to their natural uſe.

With regard to the treatment of the ſecond ſpecies of ophthalmia, I have only employed a collyrium of vitriolated zinc diſſolved in water, and mixed with vinegar and brandy; this has proved a very uſeful remedy, and has radically cured the patients in twenty or thirty days.

The third ſpecies, the inflammation of the conjunctiva, which is the ſimpleſt, but not leſs obſtinate than the former, has generally yielded to a ſolution of common ſalt in water and vinegar. I have often ſeen this diſorder cured on the coaſt of Italy, with the ſimple application of ſea-water.

Many perſons ſpeak highly of emollient and reſolving cataplaſms in all the above ſpecies of ophthalmia; but obſervation ſhews the contrary, for theſe applications relax the parts, increaſe the pain, and produce other miſchievous effects.

This is the treatment which I have employed in the military hoſpitals; and out of a thouſand patients which I have had under my care for ophthalmia, I have only to lament the total loſs of ſight in two, and of a ſingle eye each in two other patients.

*Preſervative means.*—The means of preventing ophthalmia which I have to propoſe can hardly be employed by ſoldiers, whoſe conſtant exerciſe of their profeſſion prevents them from taking care of their health, but they may be ſerviceable to others who have more leiſure and a leſs fatiguing occupation.

Firſt, a perſon ſhould avoid expoſing himſelf to the brightneſs of the ſun's light in mid-day and to the dampneſs of the night, with his head uncovered. Secondly, he ſhould bathe his eyes twice or thrice a day with cold water mixed with vinegar or lemon-juice, and this ſhould alſo be repeated whenever the eye has been irritated by duſt, ſmoke, or any ſlight blow or rubbing; and when it has been weakened by too much light, or by the evening damps, he ſhould waſh it with ſpirituous or tonic lotions. Laſtly, he ſhould avoid carefully all ſalted food, and at the ſame time keep his ſkin perſpirable, his body open, his hair rather long, and avoid ſudden cooling when he has been heated.

Theſe preſervatives are found by obſervation and experience to be very efficacious, and if employed in time they generally prevent the diſeaſe, and preſerve the eye-ſight.

---

*An Eſſay on the Structure and Formation of the Teeth in Man and various Animals, illuſtrated with copper-plates*; by ROBERT BLAKE, M. D. Dublin, 1801. 8vo. pp. 240.

THE ground work of this elaborate publication was the Author's Inaugural Diſſertation, publiſhed at Edinburgh in 1798; an occaſion which has given riſe to many of our moſt valuable Treatiſes on various branches of medical and anatomical learning.

The preſent work we conſider as containing ſo much valuable matter, matter, that a ſhort abſtract of its principal contents may not be unacceptable to our Readers. We may premiſe, that the principal object is to explain the formation, growth, and evolution of the teeth, and the changes which take place in their ſtructure, from their earlieſt rudiments in the fœtus to their perfect completion in the adult.

The firſt teeth, or thoſe of childhood, the author calls *temporary*, the ſet which ſucceeds them he terms *permanent*; he likewiſe ſubſtitutes to the term *enamel* that of *cortex striatus*, which he conſiders as more appropriate.

The firſt three chapters are employed in tracing the gradual formation of the temporary teeth from their origin to their preſent ſtate. As early as the fourth month after conception, he obſerves, the rudiments or vaſcular membranes of all the temporary teeth, and of the two anterior grinders, may be traced in each jaw. Theſe membranes, or ſacs, receive at their lower part, veſſels that depoſit within them a gelatinous ſubſtance or pulp, which ſoon becomes very vaſcular, and upon which the bony part of the tooth is afterwards formed, as on a mould. The oſſification on this pulp begins a little before birth, and always on the upper part of the future tooth or the grinding ſurfaces, and thus forms elaſtic bony ſhells, which are eaſily obſerved on examining the jaw of a new-born child. Beſides this, the ſac that inveſts the pulp ſecretes from its inner ſurface a ſoft earthy matter, which attaches itſelf to the newly-formed oſſifications, and becomes afterwards the *cortex striatus* or *enamel*. The bony part of the tooth is formed from without inwards, ſo that its external ſhell or lamina is fitted to receive the fibres of the enamel, whilſt the inner part is ſtill pulpy and not yet oſſified. During the whole time that the bony ſhell is increaſing in thickneſs, and conſequently the pulp diminiſhing in equal proportion, the connection between the two is ſo ſlight, that the ſhell may be removed from off the pulp without apparent violence, exhibiting the latter covered with a very delicate and vaſcular membrane.

The pulp has originally no proceſs correſponding with the root of the future tooth; but as the oſſification advances, the pulp extends downwards, aſſuming the ſhape of theſe proceſſes, and therefore always preceding the formation of bone, to which it appears abſolutely eſſential. When the tooth is perfect, a ſmall portion of pulp remains in the centre, forming a ſoft bed for the reception of the blood veſſels and nerves which ſupply this organ.

The membrane which depoſits the earthy matter of the enamel, ſurrounds looſely the body of the tooth, but is cloſely attached round the neck, where alſo it becomes thinner than the other part. When the enamel is perfected, this membrane, having fulfilled the purpoſe for which it was formed, is gradually abſorbed and loſt. The upper part of the gum alſo is now removed by the ſame means, and thus allows of the paſſage of the tooth up into the mouth during the proceſs of dentition, ſometimes however with conſiderable pain and difficulty to the child. Several forcible arguments prove that this this is owing to *absorption* of the refifting parts, (as Mr. Hunter well remarked) and not to the protrufive effort of the rifing tooth; and the author adds, that no refiftance to the tooth can be made by the membrane which fecretes the enamel, fince it is ftrongly attached to the neck of the tooth, and to no other part, and therefore muft partake of its motion inftead of oppofing it.

The Author obferves, that writers have made out a curious jumble in their defcription of the order in which the teeth appear during dentition; but the fact is, that there are fo many exceptions and anomalies, that no very precife general rule for expecting them can be laid down. The following, however, is here given.

"The teeth, for the moft part, appear in pairs, or the two correfponding with each other, nearly at the fame time. The firft are the middle incifores of the under jaw; in a few weeks after, the middle incifores of the upper; in a month or fix weeks after we have reafon to expect the lateral incifores of the under jaw; and in a fhort time thofe of the upper; about the twelfth or fourteenth month the anterior or fmall grinders of the under jaw appear, and frequently about the fame time thofe of the upper; about the fixteenth or twentieth month the cufpidati appear, firft in the lower jaw; and from the twentieth to the thirtieth month, the pofterior or large grinders appear in the fame order: fo that, in general, about the fecond year the twenty temporary teeth are complete."

The Author then proceeds, in the three following chapters, to defcribe the formation of the permanent teeth, the gradual enlargement of the jaw, and the appearances which attend the fhedding of the firft or temporary fet. Euftachius, Albinus, and other eminent Anatomifts, have difcovered the rudiments of feveral of the permanent teeth even in the later period of the fœtal ftate and at birth. The connexion of thefe rudiments with thofe of the temporary teeth next engages the attention of our Author, and has been the fubject of his anatomical inveftigations. The following is the refult: When the rudiments of the temporary teeth are tolerably advanced, the membrane deftined to form (in the manner before defcribed) one of the temporary teeth, fends off a new fac, which remains for fome time clofely connected with the membrane from which it took its origin, and is contained in the fame focket. This fac was obferved and defcribed by Mr. Hunter, but not its ufe; which, as the Author here obferves, and claims as his own difcovery, is to form the rudiments of the permanent tooth that is to fucceed the temporary one to which it is attached.

The gradual and fynchronous growth of each tooth is then defcribed, and the beautiful manner in which the temporary fet is made to rife into the mouth and entirely to furmount the permanent teeth, which remain deep feated till their deftined time comes on for rifing into action.

During the firft years of life, the alveolar arches continue regularly to increafe, in order to afford room for the permanent teeth; about the fourth year the two fets appear to be the moft perfect, as they are then each contained in their refpective fockets, and alfo are are ſeparated from each other by a bony partition. The ſubſequent abſorption of the neck and roots of the firſt ſet, and the method in which the permanent teeth advance partly into the ſockets of the preceding, and partly bringing their own ſocket along with them, are deſcribed with much anatomical minuteneſs.

The Author then proceeds to Comparative Anatomy, and introduces many valuable anatomical and phyſiological facts, which, however, require a conſtant reference to the plates to be well underſtood. We may juſt remark, that as the wants and habitudes of graminivorous animals differ from thoſe of the carnivorous, and as the food of the former requires much more comminution than that of the latter, a very great and important difference is obſervable in the ſtructure of the teeth of animals that feed on hard vegetable food. This conſiſts principally in the circumſtance that the enamel (or cortex ſtriatus, as the author terms it) not only inveſts the body of the tooth, but deſcends through its ſubſtance, forming therein a variety of convolutions, and therefore dividing the tooth into alternate portions or laminæ of bone and enamel. Hence, the upper or grinding ſurface is compoſed of alternate portions of unequal hardneſs; and thus, as the tooth wears away, the bony part, which is the ſofteſt, is ground down firſt, and leaves the layers of enamel always projecting above them,—a beautifulc ontrivance for enſuring that conſtant inequality of ſurface which is requiſite to comminute and divide hard bodies, a contrivance ſimilar to the notching of mill-ſtones.

The Author remarks however in the graminivorous teeth, another ſubſtance, of a hardneſs intermediate between bone and enamel, which is external to both, and therefore immediately adheres to the outer coat of enamel. This ſubſtance he terms *crusta petrosa*, and it fills up all the convolutions of the external plate of the enamel, extending ſo high as to form a conſiderable portion of the grinding ſubſtance. It appears to be of uſe, in filling up the mouth, giving compactneſs to the teeth, and ſtill further enſuring the inequality of the grinding ſurface, as it will naturally wear away faſter than the enamel, but leſs ſo than the bone. It is very eaſily diſtinguiſhed in the horſe, the cow, and large graminivorous quadrnpeds.

Several practical obſervations are added concerning the treatment of the teeth and gums during dentition, and particularly on the propriety of the common operation of lancing the gums of children to relieve the irritation ſo often attending the paſſage of the teeth into the mouth. The author objects to the frequent uſe of the lancet in this caſe, from the injury that it may often occaſion to the ſockets even of the permanent ſet. We muſt own however that the ideas which he throws out in this place are deſultory, without preciſion, and altogether unſatisfactory.

The opinions and obſervations of Mr. Hunter are canvaſſed with great freedom in the courſe of this intereſting work, and a variety of the aſſertions advanced by this late eminent anatomiſt are abſolutely contradicted.

Nine very clear and well executed plates are added, which illustrate the anatomical defcription that forms the greater part of the volume, and exhibit in a very fatisfactory manner the ftructure of the teeth in man and various other animals.

A Supplement is added, which the reader will perufe with regret, as it directly impeaches the liberality and good faith of two gentlemen, (Mr. Corfe and Mr. Home) whofe valuable and interefting papers concerning the ftructure of the elephant's teeth, publifhed in the Philofophical Tranfactions for 1799, have made an important addition to a very curious branch of Comparative Anatomy

Our readers, we are perfuaded, will readily excufe us from entering into the particulars of a perfonal difpute, and we fhall therefore conclude with expreffing our fatisfaction at the variety of curious refearch which appears on a perufal of this interefting volume.

---

*A Collection of Papers intended to promote an Institution for the Cure and Prevention of infectious Fevers in Newcastle and other populous Towns, &c. &c. By* John Clark, M. D. Newcaftle upon Tyne, p. 239, 8vo. 1802.

The fubject which has given rife to this publication appears to be the following: The Infirmary at Newcaftle, which was originally conftructed on a plan fimilar to moft of the older inftitutions of this kind, and therefore not equal in convenience and eligibility to the modern hofpitals, underwent, a year ago, a number of important improvements, and amongft others, the addition of fever-wards fufficient for the accommodation of twenty patients. When compleated, it was propofed by the editor of this publication, Dr. Clark, (a phyfician of high eminence in the town) to extend the original object of the inftitution, by converting thefe fever-wards of the Infirmary to the purpofe of a general houfe of recovery for all infectious fever which might occur in the town. This propofal it feems has met with warm oppofition, and has divided the fentiments of the inhabitants on a queftion fimilar to that which was fo warmly agitated at Manchefter, namely, How far, confiftently with general fafety, can Fever Houfes be eftablifhed in the midft of a populous city? or, in the prefent cafe, What rifk is there of the ordinary patients of the Infirmary catching the infection of fever from dedicating a detached part of the houfe to the reception of fever patients?

As the queftion of the limit to which active contagion can extend has been fo thoroughly agitated, efpecially within this laft year or two, we fhall forbear to enter particularly into the contents of this Collection of Papers. A very large part of them refers to matters purely local, the reft is made up of original correfpondence on this fubject between the Editor and feveral of our moft eminent medical practitioners who have particularly attended to the fubject of infection; among whom we notice the names of Haygarth, Currie, Ferriar, Willan, Gregory, Rutherford, &c. &c. In looking over this confufed heap of letters, memorials, refolutions of committees, and

*memoires justificatives*, we cannot help remarking that all the correfpondence from the above mentioned phyficians moft ftrongly and pointedly enforces the perfect fafety of the plan propofed by Dr. Clark, of appropriating a part of the Infirmary to a Fever Houfe for general reception; the committee however, with whom refted the determination, appeared to be of a different opinion, and the plan feems to have been negatived, or fo much modified as to be totally altered from the intention propofed. The opinions and reafoning of the medical gentlemen who oppofed Dr. Clark's plan are alfo ftated at full length, fo that the reader may have a very full view of the controverfy. The weight of opinion at leaft, if not of evidence, appears in this inftance to have been fo equally balanced, that we fee no other reafon for doubting that thofe (out of the profeffion) who were to decide the point in difpute, were enabled to make a fair eftimate of it, than a fymptom of the bitternefs of political rancour which in one place breaks out, and interferes moft unworthily (if it has interfered) with the temperate difcuffion of a very momentous queftion of general utility.

---

*A Treatise on Brown's System of Medicine, translated from the German of* H. C. Pfaff, M. D. *Professor in the University of Keil, by* John Richardson. *London*, 1802, *p.* 80. 8*vo.*

The fyftem of the celebrated John Brown, confidered merely as a fyftem, now excites but little attention in this and in its native country; our fchools for medical difputation feldom refound with the controverfies on fthenic and afthenic difeafes, excitement and debility, which fome years ago violently, but partially, agitated the medical world: the flow and filent progrefs of improvement has not however been at a ftand; the good fenfe which certainly characterifes our medical practice, has led us to employ, fomewhat more liberally than formerly, the ftimuli of Sydenham, and to engraft into our technical language the phrafeology and reafoning of Brown. Now, however, the zeal for the Brunonian hypothefis has fpread through Italy and Germany; and thofe controverfies are in full vigour there, which have fallen into partial neglect here.

The Author of the Treatife before us has made known the fyftem of Brown to his countrymen by the medium of a German tranflation, and the prefent remarks are intended as a fummary view of the leading features of the Brunonian hypothefis, pointing out the force of the excellent parts of the fyftem, and the weaknefs of its errors and deficiences.

We fhall not enter into the particular points which the learned Author argues; the objections which he urges are not new (in this country at leaft) but are well and forcibly applied; the acknowledged defects of this fyftem are, its exceffive fimplicity, which in many inftances degenerates into nakednefs and barrennefs, the want of juft difcrimination in cafes of infinite practical importance, and a fyftematical poverty in the felection and ufe of the articles of the materia medica, whereby many highly valuable fubftances are rejected.

The reader who is attached to the theoretical part of medical learning will find this an interefting effay to perufe. The tranflation is inelegant and full of *Germanisms*, but apparently accurate.

---

*A Series of Engravings, accompanied with Explanations which are intended to illustrate the Morbid Anatomy of some of the most important Parts of the Human Body; by* MATTHEW BAILLIE, M.D. F.R.S. *The ninth Fasciculis.*

THE ninth number of this highly valuable work contains eight plates, illuftrative of the moft important changes to which the female organs are fubject.

The fubject of the firft plate is ulcer of the uterus, which has generally been confidered as cancerous, and is certainly as formidable; but Dr. B. does not regard it as a genuine cancer, as the uterus does not undergo the fame changes of ftructure; it neither enlarges, nor forms any of thofe cyfts, and that kind of fungus, by which cancer is characterifed.

The difeafe begins from the cervix, and fpreads to all the neighbouring parts, and even fometimes affects the rectum and bladder.

In the fecond plate a fchirrous enlargement of the uterus is reprefented, and the nature of this fingular increafe is alfo exhibited by a tranfverfe fection.

The third plate exhibits the tubercles which fometimes form on the outer furface of the uterus, and adhere to it loofely by cellular membrane.

The important difeafe of polypus of the uterus forms the fubject of the fourth plate. It is exhibited as hanging down from the neck of the uterus, to which it is attached by a fmall peduncle, and occupying the cavity of the vagina; and the mode of operation by ligature is thus readily underftood.

Two difeafes, or accidents, of great importance to the practitioner, the prolapfus uteri, and the inverfio nteri, are reprefented in the fifth plate. In the complete prolapfus, the uterus hangs confiderably below the external labia.

A beautiful drawing of the dropfical ovarium is given in the fixth plate.

The feventh plate exhibits that rare change of ftructure in the ovarium in which it is found to contain maffes of fuetty matter, hair, rudiments of teeth or bones, and other imperfectly organized fubftances. Dr. B. having met with this in fubjects before the age of puberty, is difpofed to confider it as independant of impregnation.

The eighth and laft plate of this very important Fafciculus reprefents the very uncommon difeafe of dropfy of the Fallopian tube. In fuch cafes both the apertures are obliterated.

---

*Obfervations fur la Phthifie Pulmonaire, ou Effai fur la Mouffe d'Iflande, &c.* Par J. B. REGNAULT, M.D. 8vo. London, 1802, pp. 101.

THIS is the fame work that we recommended in our laft Number, (p. 463)

(p. 463) tranflated into French by the author, with fome additional notes and obfervations, and the fame coloured plate. Our reafon for mentioning it again is for the purpofe of anfwering a queftion which has been frequently afked fince the publication of our account of the Englifh edition, viz. "Where can the genuine plant be bought?" Dr. Regnault informs us, that he has imported a quantity, and ordered more, for the purpofe of fupplying practitioners with the real Lichen Iflandicus.

---

*Practical Observations on Vaccination, or Inoculation for the Cow-Pock.* By J. R. Coxe, M. D. &c. Illuftrated by a coloured plate, reprefenting a Comparative View of the various Stages of Vaccine and Small Pox. 8vo. pp. 152. Philadelphia, 1802.

This correct and fyftematic work is dedicated to Dr. Jenner. The Author does not confine himfelf to practical directions only for conducting Vaccination, but enters into the hiftory of the Cow-pox, and of Dr. Jenner's difcovery of its application. He appears to be familiar with all the beft works which have been publifhed in Europe on the fubject; and whenever his own experience is defective, he fupplies it by appofite references to thefe authors

